# The Small GTPase Rif Is Dispensable for Platelet Filopodia Generation in Mice

**DOI:** 10.1371/journal.pone.0054663

**Published:** 2013-01-24

**Authors:** Robert Goggs, Joshua S. Savage, Harry Mellor, Alastair W. Poole

**Affiliations:** 1 School of Physiology and Pharmacology, University of Bristol, Bristol, United Kingdom; 2 School of Biochemistry, University of Bristol, Bristol, United Kingdom; Karolinska Institutet, Sweden

## Abstract

**Background:**

Formation of filopodia and other shape change events are vital for platelet hemostatic function. The mechanisms regulating filopodia formation by platelets are incompletely understood however. In particular the small GTPase responsible for initiating filopodia formation by platelets remains elusive. The canonical pathway involving Cdc42 is not essential for filopodia formation in mouse platelets. The small GTPase Rif (RhoF) provides an alternative route to filopodia generation in other cell types and is expressed in both human and mouse platelets.

**Hypothesis/Objective:**

We hypothesized that Rif might be responsible for generating filopodia by platelets and generated a novel knockout mouse model to investigate the functional role of Rif in platelets.

**Methodology/Principal Findings:**

Constitutive *RhoF^−/−^* mice are viable and have normal platelet, leukocyte and erythrocyte counts and indices. *RhoF^−/−^* platelets form filopodia and spread normally on various agonist surfaces in static conditions and under arterial shear. In addition, *RhoF^−/−^* platelets have normal actin dynamics, are able to activate and aggregate normally and secrete from alpha and dense granules in response to collagen related peptide and thrombin stimulation.

**Conclusions:**

The small GTPase Rif does not appear to be critical for platelet function in mice. Functional overlap between Rif and other small GTPases may be responsible for the non-essential role of Rif in platelets.

## Introduction

On encountering damaged endothelium, quiescent platelets become activated and undergo dramatic alterations in shape, due principally to reorganization of the actin cytoskeleton. Among the earliest events is the extension of filopodia [1]. These thin (0.1–0.3 µm) finger-like membrane protrusions enclosing tight parallel bundles of filamentous actin (F-actin) increase the potential for contact between platelets and extracellular matrix and for interactions with other cells [2]. The actin rearrangements that underlie these shape change events are regulated by Rho-type (Ras-homology) G-proteins. These small GTPases have prominent roles in regulation of cell shape, polarity and motility and in platelets, are involved in formation of focal adhesions (RhoA) [3], lamellipodia (Rac) [4] and filopodia (Cdc42) [5], [6], [7].

Filopodia formation requires nucleation and polymerization of F-actin filaments. The canonical pathway involves Cdc42, which activates the Arp2/3 protein complex and interacts with nucleation promoting factors of the Wiskott-Aldrich Syndrome protein (WASp) family, the membrane deforming protein IRSp53 and the formin protein mDia2 [8]. Cdc42-independent pathways to filopodia formation likely exist in platelets however. Megakaryocyte- and platelet-specific deletion of the *Cdc42* gene in mice results in mild macrothrombocytopenia and shortened platelet lifespan, but *Cdc42^−/−^* platelets form filopodia with normal morphology on fibrinogen coated surfaces [7]. Cdc42 is not fully redundant in platelets however because filopodia formation on von Willebrand factor (VWF) is defective in *Cdc42^−/−^* platelets.

The small GTPase Rif (RhoF) was identified from partial cDNA sequences during a search for novel Rho-family GTPases [9]. Messenger RNA for Rif has since been identified in human megakaryocytes [10] and in human and mouse platelets [11]. Ectopic Rif expression in cultured cells promotes formation of long, thin, flexible, actin-rich protrusions [12], [13]. Rif is involved in dendritic spine morphogenesis [14] and also induces stress fibre formation in HeLa cells [15]. In various cell types in culture, Rif generates filopodia through interactions with the formin proteins mDia1 [15], [16] and mDia2 [17]. Formin proteins directly nucleate actin filament polymerization at filopodial tips [18], [17] and as such provide a pathway to filopodia formation that is independent of Cdc42 and of the Arp2/3 protein complex. We hypothesized that Rif acting in concert with formin proteins expressed in platelets [19] might provide an alternative pathway for platelet filopodia generation.

In the present study, we used a novel constitutive *RhoF* gene deletion mouse model to investigate the role of Rif in platelets. The *RhoF^−/−^* mouse is viable and shows no overt phenotype. Contrary to our hypothesis, *RhoF^−/−^* platelets form filopodia and spread normally on a range of agonist surfaces both in static conditions and under arterial shear which suggests that Rif is not required for shape change or filopodia formation by platelets. Similarly, Rif appears to be dispensable for other platelet functions including integrin activation, aggregation and secretion from both dense and α-granules. Functional redundancy between Rif and other small GTPases may provide an explanation for these findings.

## Methods

### Ethics Statement

Mice were bred and maintained in the University of Bristol animal facility in accordance with United Kingdom Home Office regulations and all procedures were undertaken in accordance with the Animals (Scientific Procedures) Act (ASPA), 1986 (project license numbers: 30/2386 and 30/2908). The local research ethics committee at the University of Bristol, UK approved the use of mice for this study.

Blood was drawn into 4% trisodium citrate (1∶9) by descending vena cava puncture of mice humanely euthanized by exposure to rising CO_2_ gas concentrations per Schedule 1 of ASPA, 1986. All efforts were made to minimize suffering.

Blood was obtained from healthy drug-free volunteers in accordance with approved guidelines and with ethical approval granted by the local research ethics committee, University Hospitals Bristol NHS Foundation Trust (E5736). Written, informed consent was obtained in accordance with the Declaration of Helsinki.

### Materials

Except where specified all chemicals were obtained from Sigma (Poole, Dorset, UK).

Human Rif antibodies were generated in house (HM lab). Mouse Rif antibodies (ab81024) were from Abcam (Cambridge, UK). Antibodies against Rac (2465) and Cdc42 (2462) were from Cell Signaling Technology (Danvers, MA, USA). RhoA (26C4) and RhoG antibodies (1F3 B3 E5) were obtained from Santa Cruz Biotechnology (Insight Biotechnology, Wembley, UK). Phycoerythrin (PE) conjugated JON/A (anti-active α_IIb_β_3_) and fluorescein isothiocyanate (FITC) conjugated JAQ-1 (anti-GPVI), Xia.G5 (anti-GPIbα) and WugE9 (anti-CD62-P) antibodies were from Emfret Analytics (Würzburg, Germany). FITC-conjugated MWReg30 (anti-CD41) antibody and its isotype-matched control, FITC-conjugated rat IgG1, were from AbD Serotec (Kidlington, Oxfordshire, UK). DiOC6 was from Axxora (Nottingham, UK). NuPAGE LDS sample buffer was obtained from Invitrogen (Carlsbad, CA, USA). Secondary HRP-conjugated antibodies, enhanced chemiluminescence solutions and autoradiography film were obtained from Amersham Inc. (Little Chalfont, Buckinghamshire, UK). Western blotting solutions and equipment were from Bio-Rad (Hemel Hempstead, UK) and Fermentas (St. Leon-Rot, Germany). Luciferin-luciferase (Chrono-Lume Reagent) and horm collagen (Chrono-Par Collagen) were from Chrono-Log (Labmedics, Manchester, UK). The snake venom protein botrocetin was a generous gift from Prof. Robert Andrews (Australian Centre for Blood Diseases, Monash University, Australia). The GPVI specific agonist CRP (cross-linked collagen-related peptide) was synthesized by Prof. Richard Farndale (Department of Biochemistry, University of Cambridge, UK). Mowiol, von Willebrand’s Factor (VWF) and D-phenylalanyl-prolyl-arginyl chloromethyl ketone (PPACK) dihydrochloride were from Calbiochem (Merck Chemicals, Nottingham, UK). Polyvinylidene difluoride (PVDF) transfer membrane (Immobilon P) was from Millipore (Merck Chemicals, Nottingham, UK).

### Generation and Genotyping of *RhoF^−/−^* Mice

The *RhoF^−/−^* mice were derived by Geneta (University of Leicester, UK) using gene targeted embryonic stem (ES) cells generated by Taconic (Köln, Germany). Littermate *RhoF^+/+^* mice (henceforth known as wild-type) were used as controls. Mice were maintained on a C57/Bl-6 background. Genotyping of *RhoF^−/−^* mice was undertaken using duplex PCR reactions to enable identification of both mutant and wild-type alleles. The three primers used were: AACGCACAGAAGGCAGAGGC, CCTCCACAGCCCCAGTCCAA and CCTGTTGGTGCAATCGTGGCG. PCR products were analysed on 2% agarose gels containing ethidium bromide 1∶25,000 against appropriate molecular weight markers (GeneRuler 100 bp DNA Ladder, Fermentas) under UV transillumination.

### Preparation of Mouse Platelets

Mice, 8 to 12 weeks of age, of mixed gender were used for all experiments. Complete blood counts were conducted using a Pentra ES60 hematology analyser (Horiba Medical, Northampton, UK) prior to platelet preparation and counts adjusted for anticoagulant volume. Washed platelets were prepared as described previously [20]. In brief, blood was diluted with 800 µl of modified Tyrode’s-HEPES buffer (135 mM NaCl, 3 mM KCl, 10 mM HEPES, 5 mM glucose, and 1 mM MgCl2.6 H_2_O, pH 7.3) and centrifuged at 180 g for 6 min at room temperature. Platelet-rich plasma was removed, and platelets were isolated by centrifugation at 550 g for 10 min in the presence of PGE_1_ (140 nM), indomethacin (10 µM) and apyrase (0.02 U/ml). Pelleted platelets were resuspended in Tyrode’s-HEPES buffer, counted using a Z1 Coulter particle counter (Beckman Coulter, High Wycombe, UK), diluted to the required density in modified Tyrode’s-HEPES buffer and rested for 30 min at 30°C in the presence of 10 µM indomethacin and 0.02 U/ml apyrase prior to stimulation.

### Preparation of Human Platelets

Indomethacin (10 µM) and apyrase (0.02 U/ml) were added to PRP and throughout subsequent preparation steps, as described previously [21].

### Immunoblotting

Washed platelets (2×10^8^ cells/ml) were lysed in NuPAGE LDS sample buffer supplemented with 50 mM dithiothreitol and heated at 95°C for 5 minutes. Proteins were separated by electrophoresis using 10–15% Bis-Tris polyacrylamide gels (Mini-Protean 3, BioRad) against known molecular weight markers (PageRuler Plus, Fermentas). Proteins were transferred onto PVDF membranes which were blocked in 5% bovine serum albumin (BSA) in Tris-buffered saline/Tween-20 (10 mM Tris, 150 mM NaCl, 0.1% Tween-20). Membranes were probed with primary antibodies and appropriate horseradish peroxidase conjugated secondary antibodies. Proteins were detected by enhanced chemiluminescence using autoradiography film.

### Subcellular Morphology

Subcellular morphology of wild-type and *RhoF^−/−^* platelets was analysed by transmission electron microscopy (TEM). Ultrathin counterstained sections were prepared as previously described [20]. Mouse platelets were pelleted from platelet rich plasma as described above. The pellet was fixed in 2.5% glutaraldehyde in 0.1 M phosphate buffer (PB) (pH 7.4), washed in PB and then incubated with 1% osmium tetroxide in PB for 30 minutes. After washing in PB and deionized water, the pellet was incubated for 30 minutes with 3% uranyl acetate in deionized water. After washing with deionized water, the pellet was dehydrated in a graded series of increasing ethanol concentrations (70%, 80%, 90%, 96% and 100% with each step lasting 10 minutes). The pellet was then incubated with Epon 812 for 2 hours at room temperature, the Epon was refreshed and then hardened overnight in a 60°C oven. Sections were imaged with an FEI Tecnai G2 Spirit T12 BioTwin Transmission Electron Microscope and images captured at x2800 magnification using an FEI Eagle 4K CCD camera. To determine the dense-granule and α-granule content, total numbers of granules in equivalent-sized fields of view were quantified manually using ImageJ 1.45e (NIH, http://rsbweb.nih.gov/ij/). Granule numbers are expressed as granules per cell per section.

### Turbidometric Aggregometry and ATP Secretion

Platelet aggregation studies were conducted using 245 µl aliquots of washed platelets at 2×10^8^ cells/ml in a two-channel Born lumi-aggregometer (560-VS, Chrono-Log, Havertown, PA) using proprietary software (Aggro/Link 5.6.3, Chrono-Log). Aggregation was initiated using final concentrations of 0.2–5 µg/ml CRP or 0.01–1 U/ml bovine α-thrombin at 37°C, stirred at 1000RPM. Where appropriate, ATP secretion was measured simultaneously with aggregation using a luciferin-luciferase assay with 2 nmol ATP standard added after each aggregation to calibrate the assay.

### Platelet Spreading Assays

Glass coverslips were coated with 50 µg/ml CRP, 100 µg/ml fibrinogen or 10 µg/ml VWF in Tyrode’s-HEPES buffer overnight at 4°C then blocked with 2% fatty-acid free BSA for 1 h at room temperature. Slides were washed with Tyrode’s-HEPES buffer before and after blocking. Aliquots of 100 µl washed platelets at 3×10^7^ cells/ml were applied to coverslips and platelets allowed to adhere for predefined time periods. For VWF coated surfaces, platelets were allowed to adhere in the presence of 5 µg/ml botrocetin. Where indicated, platelets were stimulated with bovine α-thrombin (1 U/ml) during adhesion. Excess sample was then removed; unbound cells carefully washed away and adhered platelets fixed with 4% paraformaldehyde for 15 minutes at room temperature. After washing, adherent cells were permeabilized for 15 minutes with 0.05% Triton X-100 in PBS and stained with FITC-conjugated phalloidin for 1 h. Coverslips were mounted onto microscope slides with Mowiol-DABCO and imaged with an inverted fluorescence microscope (DM-IRB, Leica Microsystems, Wetzlar, Germany). Images were taken using a microscope-mounted CCD (ORCA-ER-1394, Hamamatsu, Welwyn Garden City, UK) and dedicated software (Volocity, PerkinElmer, Waltham, USA) and analysed in a blinded fashion with ImageJ. For live cell differential interference contrast (DIC) imaging, uncoated multichamber plastic slides (8-well μ-Slide, Ibidi, Thistle Scientific, Glasgow, UK) were coated as above. Slides containing 170 µl per well Tyrode’s-HEPES buffer were equilibrated to 37°C on the microscope stage prior to addition of aliquots of 30 µl of 2×10^8^ cells/ml to each well (final concentration 3×10^7^ cells/ml). Images were collected at 1 frame/s for 20 minutes and the stacked images compiled into video files using ImageJ.

### Flow Cytometry Assays

For all flow cytometry assays, platelets were diluted to 2×10^7^ cells/ml in Tyrode’s-HEPES buffer. All flow cytometry experiments were conducted in 5 ml polystyrene round-bottomed tubes (BD, Oxford, UK) under non-stirring conditions. For determination of expression of surface glycoproteins, 48 µl aliquots of platelets were incubated with 2 µl FITC-conjugated rat anti-mouse antibodies against CD41, GPVI and GPIbα for 10 minutes at room temperature prior to fixation. Non-specific binding was controlled using isotype-specific antibodies. Surface glycoprotein determinations were run in duplicate and the median values used for calculations. For determination of surface expression of P-selectin and activation of integrin α_IIb_β_3_, 35 µl aliquots of platelets were incubated with 5 µl agonist at appropriate concentrations for 10 minutes in the presence of 5 µl of the PE-conjugated activation state specific antibody and 5 µl FITC conjugated anti-CD62-P antibody prior to fixation. For determination of intracellular F-actin levels, 45 µl aliquots of platelets were incubated with 5 µl agonist at appropriate concentrations for 10 minutes (dose-response) or for the indicated time (time courses) prior to fixation. Platelets were then permeabilized with 0.05% Triton X-100 in PBS and stained using 2 µM FITC-phalloidin. For all flow cytometry assays, platelets were fixed with 50 µl 4% paraformaldehyde in PBS, diluted with 200 µl PBS and analysed using a flow cytometer (FACS Canto II, BD) with proprietary software (FACS Diva, BD). Platelets were identified by forward and side-scatter properties. For dual colour experiments, compensation controls were produced using FITC and PE stained beads (Calibrite Beads, BD) and applied to all fluorescence intensity values. At least 10,000 platelet-gate events were collected per experiment.

### Whole Blood Flow Chamber Assays

*In vitro* thrombus formation assays were performed as previously described [22]. Mouse blood anticoagulated with sodium citrate collected as above, with additional heparin (2 U/ml) and PPACK (40 µM) was labelled with DiOC6 (1 µM) and passed over immobilized collagen (50 µg/ml) or fibrinogen (100 µg/ml) through a parallel plate perfusion chamber at a shear rate of 1000 s^−1^ for 3 min. Phase-contrast and fluorescence images were captured at 2 frames per second with a BX51WI microscope (Olympus UK, Southend-on-Sea, UK) using a 40x water dipping objective, a Rolera-XR digital camera and QCapture software (QImaging, Surrey, BC, Canada). Coverslips were then washed with buffer to remove non-adherent cells and 30 random images then collected for each experiment. Surface coverage during flow and post washing was analysed using ImageJ. Immediately after some experiments, cells adhered to coverslips were fixed by flowing 500 µl of 4% paraformaldehyde through the chamber at 1000 s^−1^. Coverslips were then carefully removed from chambers, mounted onto microscope slides and imaged by DIC microscopy as described above. The number of filopodia per platelet and the length of filopodia on 100 cells were then analyzed. Only cells with visible filopodia were included in analyses and any overlapping cells and cells along the edges of the image were excluded. Images were analyzed in a blinded fashion, working from right to left across the image using a grid overlay. Filopodia lengths were measured from the visible tip to the base at the boundary of the cell body using the segmented line tool on ImageJ. True lengths were calculated using the pixel ratio of the CCD corresponding to the 100x objective lens used (1 pixel = 0.07 µm).

### Statistics

Unless otherwise stated data are presented as mean ± SEM and statistical significance was determined by paired Student’s t-test, performed using Prism 5.0 (GraphPad Software, San Diego, CA, USA). P<0.05 was considered significant.

## Results and Discussion

### Generation of a *RhoF* Knockout Mouse Line

**Figure 1:**
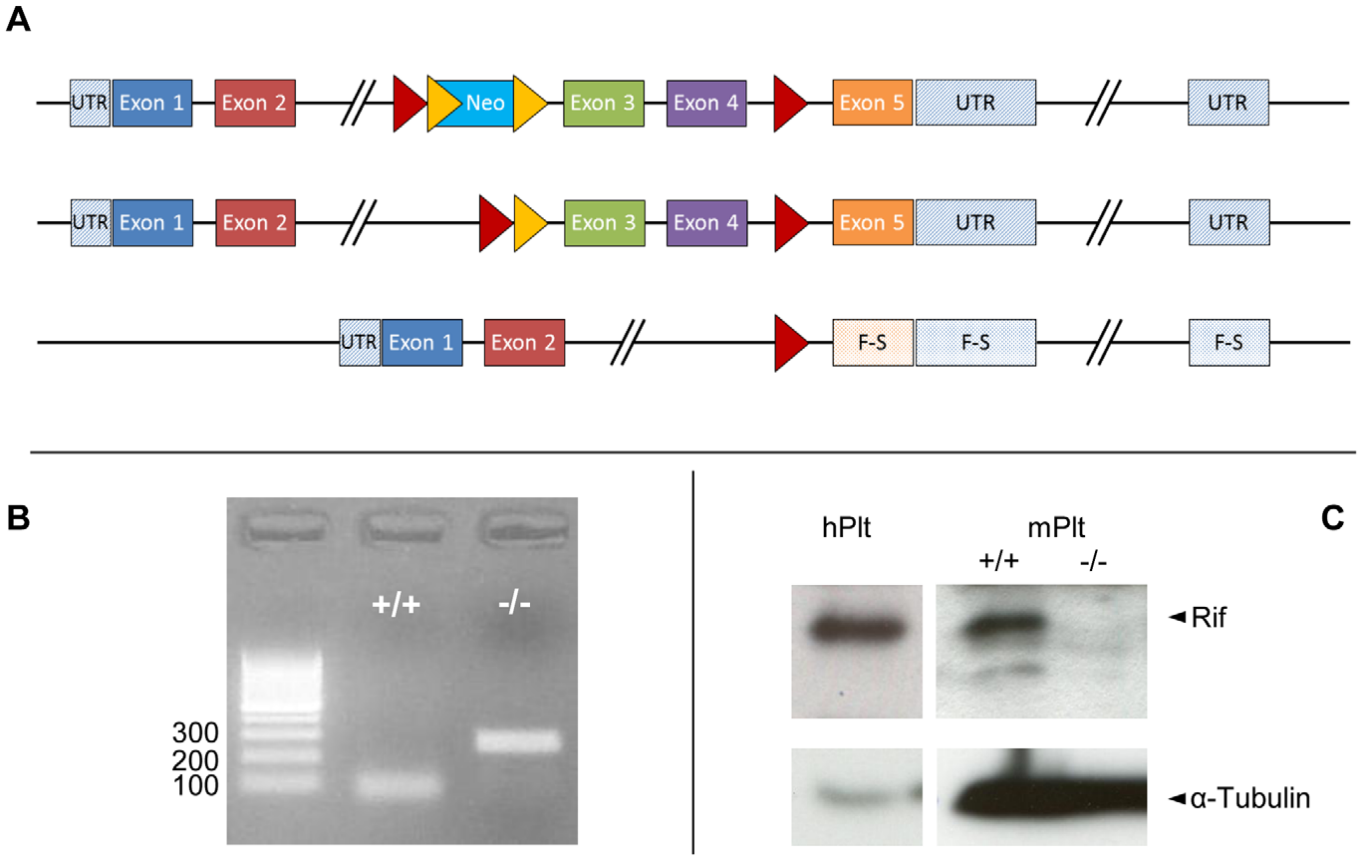
Rif is expressed in human and mouse platelets and is ablated in platelets from a constitutive *RhoF^−/−^* mouse. (**A**) Generation of the *RhoF^−/−^* mouse employed a targeting strategy which permitted generation of conditional or constitutive knockout (KO) mice. Exons 3 and 4 of the *RhoF* gene were flanked by LoxP sites and a *Neo* selection marker was inserted flanked by Frt sites such that deletion of exons 3 and 4 caused a frameshift mutation resulting in loss of function. Initially a conditional ready KO was generated after *in vivo* Flp-mediated removal of the selection marker. A constitutive KO was then generated after Cre-mediated deletion of exons 3 and 4. Red triangles represent LoxP sites, yellow triangles represent Frt sites. Abbreviations: untranslated region (UTR), frameshift (F-S). (**B**) The *RhoF^−/−^* mice genotyping strategy employed a three-primer duplex PCR reaction which generated a wild-type (+/+) product 100 bp long and a *RhoF^−/−^* product 250 bp long and enabled identification of both wild-type and *RhoF^−/−^* mice. (**C**) Analysis of Rif expression in human platelets (hPlt) and in wild-type (*RhoF^+/+^*) and Rif null (*RhoF^−/−^*) mouse platelets (mPlt) by Western blot demonstrating expression in human and wild-type mouse platelets and ablation of Rif protein expression in platelets from *RhoF^−/−^* mice.

To evaluate the function of Rif in platelets, the gene encoding this protein (*RhoF*) was disrupted by homologous recombination in ES cells, which were then used to generate gene-targeted mice by standard techniques (Fig. 1A). RhoF^Frt/Frt^ mice were crossed with mice expressing Flp recombinase under a ROSA26 promoter and the resulting offspring then crossed with mice expressing Cre recombinase under a CMV promoter. Genotyping of these mice using a duplex PCR reaction generated a wild-type product 100 bp long and a *RhoF^−/−^* product 250 bp long which enabled identification of both wild-type and *RhoF^−/−^* mice (Fig. 1B). The constitutive *RhoF* ^−/−^ mice generated were viable and healthy with no external phenotypic abnormalities. Mice were obtained in their expected Mendelian ratios, p = 0.494 (Chi square test). Of 208 offspring of heterozygote pairings genotyped, 57 were wild-type (27.4%), 109 were heterozygous (52.4%) and 42 were *RhoF* ^−/−^ (20.2%). Expression of Rif in human and mouse platelets, and effective elimination of Rif protein (23.6kDa) in *RhoF* ^−/−^ mice platelets was confirmed by immunoblotting (Fig. 1C).

### Hematologic Indices, Platelet Granule Numbers and Surface Glycoprotein Expression are Normal in *RhoF* ^−/−^ Platelets

**Figure 2:**
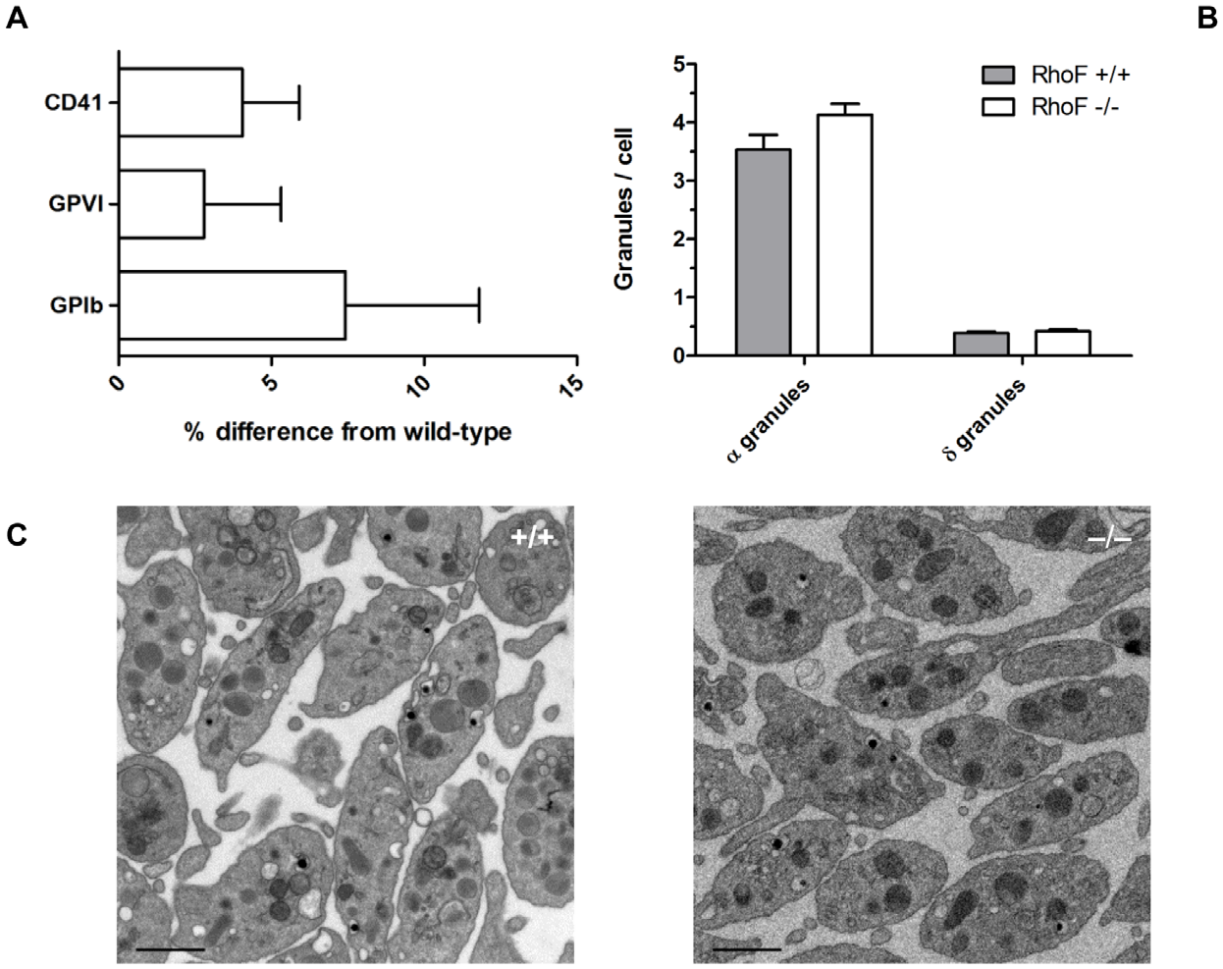
Platelets from *RhoF^−/−^* mice have normal surface glycoprotein expression, normal granule numbers and normal ultrastructure. (**A**) Expression of surface glycoproteins on *RhoF^−/−^* platelets was quantified by flow cytometry. Washed platelets were incubated with FITC labelled antibodies at saturating concentrations for 10 minutes at room temperature, then fixed and analysed by flow cytometry. Data are presented as mean ± SEM of the percentage difference from wild-type controls (n = 16). (**B**) Quantification of alpha and dense granule numbers per cell per thin section examined. Data are presented as mean ± SEM of 3 mice per group. (**C**) Representative transmission electron microscopy images of resting wild-type and *RhoF^−/−^* platelets. Scale bars represent 1 µm.

Complete blood counts conducted on *RhoF* ^−/−^ mice and on littermate controls suggest that hematopoiesis in *RhoF* ^−/−^ mice occurs normally. *RhoF* ^−/−^ mice have comparable erythrocyte, leukocyte and platelet counts to wild-types and platelets and erythrocytes from these mice are normal in size (Table 1). In contrast, megakaryocyte and platelet specific deletion of RhoA [23] or Cdc42 [7] causes macrothrombocytopenia. This implies that RhoA and Cdc42 are both required for normal proplatelet production whereas Rif is not essential for this process. Surface expression of the α_IIb_ subunit of the fibrinogen receptor (CD41), the collagen receptor GPVI and the GPIbα subunit of the VWF receptor GPIb-IX-V complex were equivalent in *RhoF* ^−/−^ platelets and wild-types (Fig. 2A), consistent with the conditional Cdc42 null mouse. Analysis of thin section transmission electron microscopy images reveals that platelets from *RhoF* ^−/−^ mice have normal ultrastructure, are equivalent in size to wild-type mouse platelets and have equivalent numbers of both alpha and dense granules (Fig. 2B, C). Thus, *RhoF* ^−/−^ mouse platelets afforded us an ideal opportunity to study platelet function in the selective absence of Rif.

**Table 1:** Summary hematologic data for wild type and *RhoF^−/−^* mice.

	*RhoF^+/+^*			*RhoF^−/−^*			
	Mean	SD	n	Mean	SD	n	p value
Plt count (x10^3^/µl)	849	97	42	799	126	30	ns
MPV (µm^3^)	5.20	0.15	42	5.24	0.16	30	ns
Pct (%)	0.44	0.05	42	0.42	0.06	30	ns
WBC count (x10^3^/µl)	9.72	3.31	42	11.76	4.05	30	ns
RBC count (x10^3^/µl)	10.28	0.43	42	10.08	0.57	30	ns
Hct (%)	49.67	2.30	42	49.44	2.41	30	ns
MCV (µm^3^)	48.33	0.98	42	49.03	1.52	30	ns

### Platelet Filopodia Formation and Spreading is Unaffected by Loss of Rif

**Figure 3:**
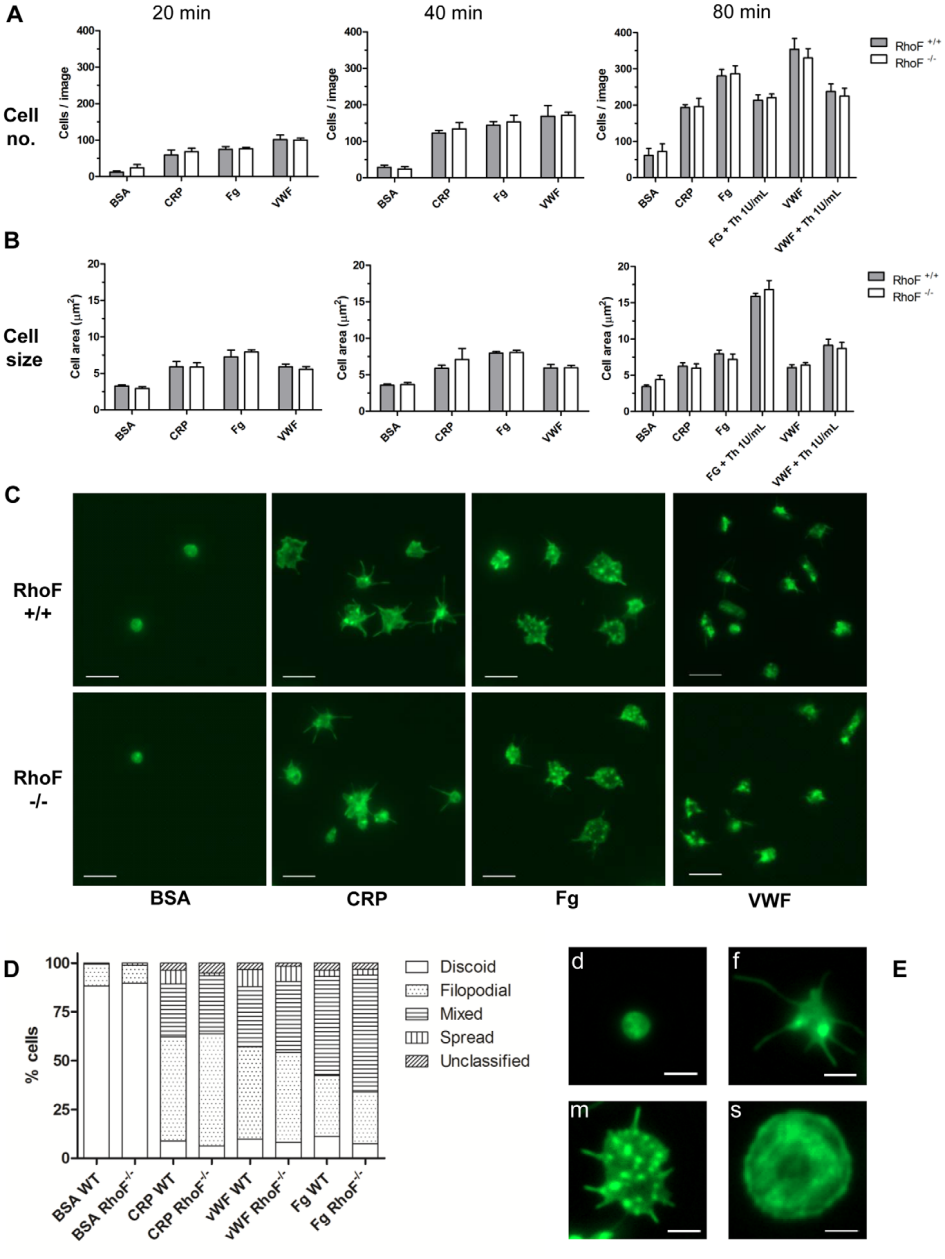
Platelets from *RhoF^−/−^* mice adhere to and spread on agonist surfaces under static conditions normally. (**A**) Aliquots of washed platelets were allowed to adhere to and spread on fibrinogen (Fg), collagen related peptide (CRP) and von Willebrand Factor (VWF) coated surfaces. For VWF coated surfaces, platelets were allowed to adhere in the presence of 5 µg/ml botrocetin. Fatty-acid free BSA coated surfaces were used as negative controls. Co-stimulation with α-thrombin (1 U/ml) was also undertaken as indicated. Numbers of cells adherent per x63 field of view were quantified at the three indicated time points. (**B**) The average area of at least 500 adherent cells was quantified per agonist surface and 100 cells quantified per control. Data are presented as mean ± SEM of at least 4 mice per group. (**C**) Representative images of adherent cells on agonist and control surfaces at 20 minutes. Scale bars represent 5 µm. (**D**) Platelet morphology at 20 minutes was visually assessed and 100 cells per experiment assigned to one of 5 categories (discoid, filopodial, mixed, spread and unclassified). Data are presented as stacked bars representing the mean percentages of cells in each category from at least 4 mice per group. (**E**) Example images of discoid (d), filopodial (f), mixed (m) and spread (s) platelets corresponding to the morphology classifications represented in Fig. 3D. Unclassified cells were those platelets with morphologies that could not be assigned to any of these four groups. Scale bars represent 2 µm.

We hypothesized that filopodia formation by platelets lacking Rif would be defective and examined the ability of *RhoF* ^−/−^ platelets to adhere to and to spread on extracellular matrix proteins including collagen and VWF and on agonist surfaces such as fibrinogen. Simultaneously these assays test the integrity of outside-in signalling from integrin α_IIb_β_3_ which binds both fibrinogen and VWF. Static adhesion assays show that *RhoF* ^−/−^ platelets adhere in equivalent numbers and spread to an equivalent degree to wild-type platelets on these surfaces (Fig. 3A, B). Morphological assessment of these cells at various time-points suggests that *RhoF* ^−/−^ platelets are not dissimilar to wild-type platelets (Fig. 3C, D) on any surface. Real-time visualisation of wild-type and *RhoF* ^−/−^ platelets by DIC video microscopy (Videos S1–S6) confirms that *RhoF* ^−/−^ platelets form filopodia and lamellipodia over equivalent time periods and are also able to produce membrane ruffles as do wild-type platelets.

Consistent with observations of normal filopodia formation and spreading by *RhoF* ^−/−^ platelets were evaluations of actin turnover in these cells. Estimation of the levels of F-actin in platelets by FITC-phalloidin staining of fixed, permeabilized cells suggests that F-actin formation in *RhoF* ^−/−^ platelets occurs to an equivalent level (Fig. S1A) and over a similar time course to that of wild-type platelets following agonist stimulation (Fig. S1B).

### *RhoF* ^−/−^ Platelets Show Normal Integrin α_IIb_β_3_ Activation and Aggregation

**Figure 4:**
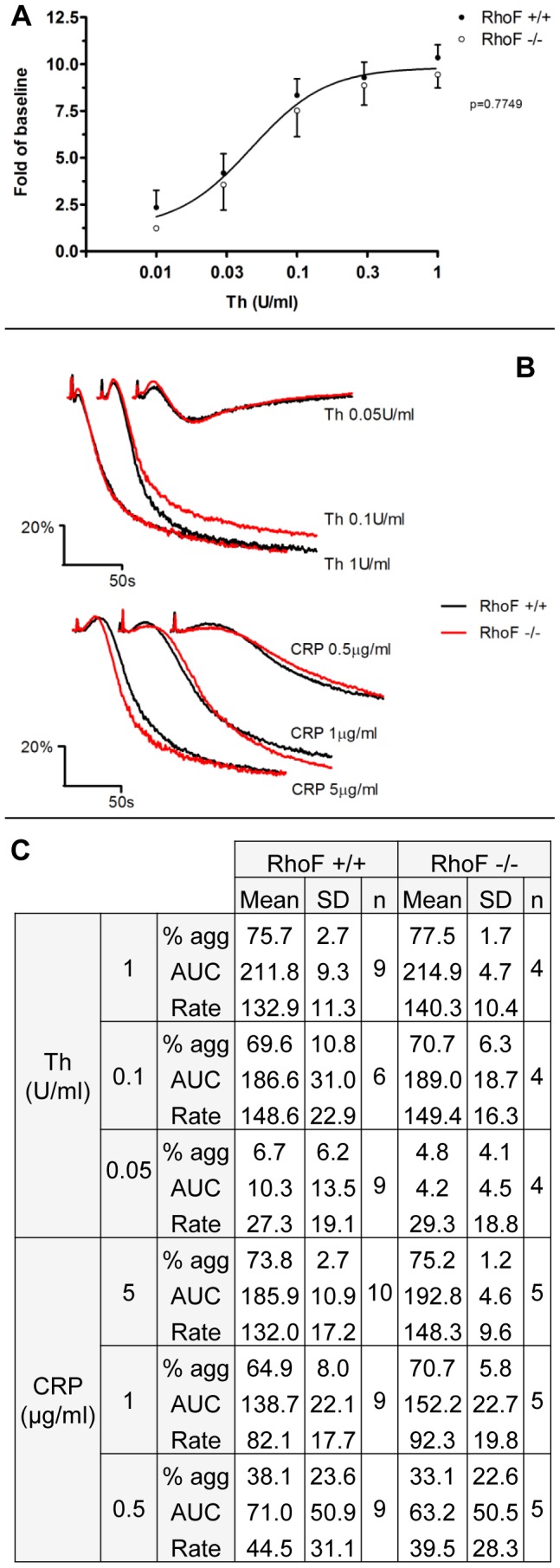
Integrin activation and light transmission aggregometry of *RhoF^−/−^* platelets is normal in response to thrombin and CRP. (**A**) Dose-response curves for activation of integrin α_IIb_β_3_ in response to thrombin stimulation as indicated by binding of the activation state specific antibody JON/A. No significant differences were identified between the fitted curves of wild-type and *RhoF^−/−^* platelets, suggesting that inside-out signalling to the integrin downstream of PARs does not require Rif. Data are presented as mean ± SEM of at least 5 mice per group. (**B**) Light transmission aggregation of *RhoF^−/−^* platelets in response to CRP is normal over a range of concentrations indicating that Rif is not required for signalling downstream of GPVI in mice. Similarly the aggregation responses of *RhoF^−/−^* platelets following thrombin stimulation are not significantly different from those of wild-type controls. Aggregation traces are representative of at least 4 mice per group. (**C**) Summary aggregation data comparing the three derived aggregation parameters, maximum aggregation at 3 minutes (% agg); the area under the aggregation curve to 3 minutes (AUC) and the maximum rate of aggregation (Rate) for *RhoF^−/−^* platelets and wild-type controls.

The potential role of Rif in inside-out signalling to integrin α_IIb_β_3_ was evaluated by flow cytometric assessment of levels of activated integrin following thrombin stimulation (Fig. 4A). Construction of a dose-response curve for integrin activation by thrombin enabled evaluation of signalling downstream of both G_q_ (high concentrations) and G_13_ (low concentrations). Binding of the activation state specific antibody JON/A in *RhoF^−/−^* platelets was equivalent to wild-type, indicating that Rif is not required for inside-out signalling to α_IIb_β_3_ downstream of protease activated receptors (PARs). Similarly, aggregation responses of *RhoF^−/−^* mice to a range of concentrations of thrombin and CRP were similar between *RhoF^−/−^* mice and wild-type controls, again suggesting that inside-out signalling to the fibrinogen receptor downstream of PARs and of the collagen receptor GPVI are normal in mice platelets lacking Rif (Fig. 4B, C).

### *RhoF* ^−/−^ Platelets Secrete Normally from Both Alpha and Dense Granules

**Figure 5:**
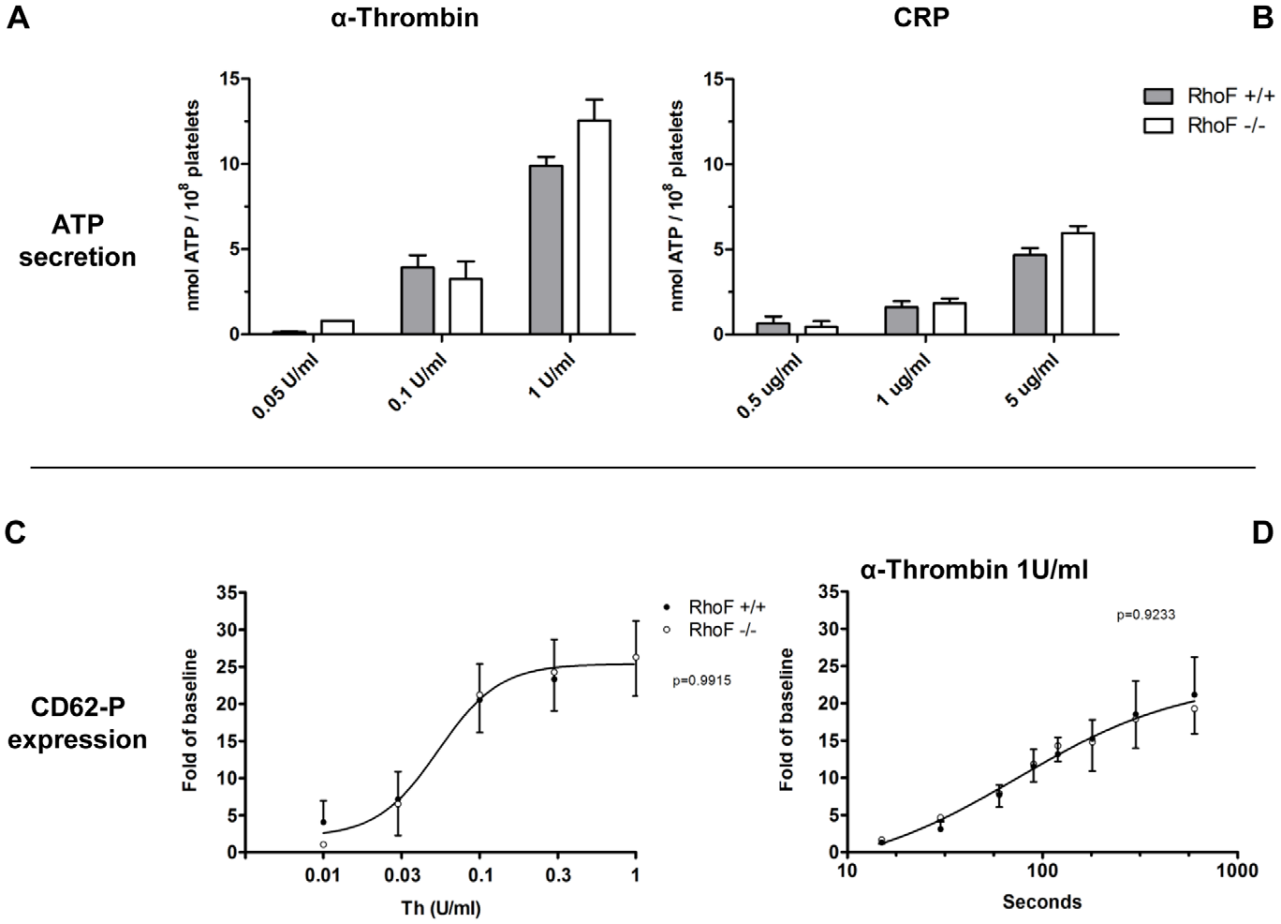
Release of ATP is normal in *RhoF^−/−^* mice in response to thrombin (**A**) and CRP (**B**) showing that Rif is not required for dense granule secretion. Data are presented as mean ± SEM of at least 4 mice per group. Similarly, expression of P-selectin induced by thrombin stimulation is normal over a range of concentrations (**C**) and follows a similar time course to that in wild-type controls (**D**). Rif does not therefore appear to be required for the cytoskeletal rearrangements required for alpha granule secretion in mouse platelets. Data are presented as mean ± SEM of at least 3 mice per group.

Although Rif appears to be dispensable for filopodia formation and cell spreading in platelets, we investigated its role in platelet granule secretion; since increased secretion in *Cdc42^−/−^* platelets in response to G-protein and tyrosine kinase coupled agonists has been previously reported [7]. Dense granule secretion of ATP, monitored by luminometry, was stimulated by a range of concentrations of CRP and thrombin. No difference in ATP secretion was observed between wild-type and *RhoF^−/−^* platelets at any concentration tested (Fig. 5A), indicating that Rif is not required for the secretion of dense granule content in platelets.

Next, we addressed whether Rif has a role in α-granule secretion by evaluating P-selectin expression on the platelet surface using flow cytometry. P-selectin expression induced by various concentrations of thrombin was not significantly affected in *RhoF^−/−^* platelets compared to wild-type (Fig. 5C), suggesting that Rif is not required for α-granule secretion in platelets. In addition, analysis of the time course of α-granule secretion indicated that the rate of P-selectin expression was not different between wild-type and *RhoF^−/−^* platelets (Fig. 5D).

Taken together, these data show that Rif is not essential for granule secretion by mouse platelets. It is possible that Rif genuinely plays no role in granule secretion in platelets and that granule secretion is regulated by other Rho GTPases including negative regulation by Cdc42 [7] and positive regulation by RhoA [23]. Similarly the finding that *RhoF^−/−^* platelets contain normal numbers of both α-granules and dense granules (Fig. 2B) suggests that Rif is not required to orchestrate actin rearrangements for vesicle trafficking in megakaryocytes either.

**Figure 6:**
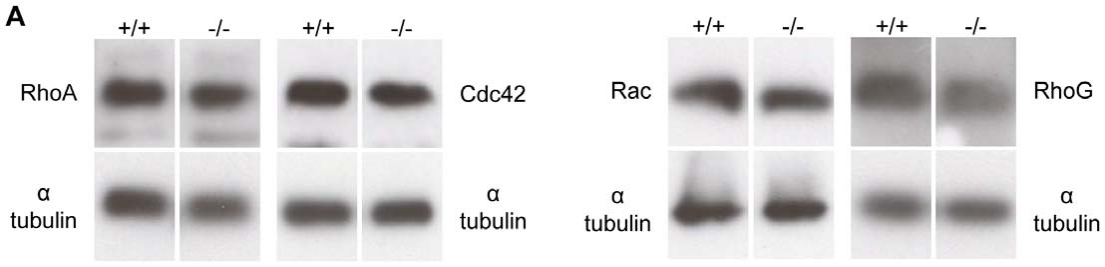
Expression of other small GTPase proteins is normal in *RhoF^−/−^* mice. (**A**) Analysis of expression of the small GTPases Rac, RhoA, Cdc42 and RhoG in platelets from *RhoF^−/−^* mice. α-tubulin was used as a loading control.

We considered the possibility that other small GTPases might be over-expressed in *RhoF^−/−^* platelets, sufficient to explain the lack of a phenotype in the *RhoF^−/−^* mice. To evaluate this, expression of RhoA, Rac, Cdc42, and RhoG in *RhoF* ^−/−^ platelets was estimated by immunoblotting (Fig. 6A). No up-regulation of expression of any of these GTPases was detected in *RhoF^−/−^* platelets however, suggesting that the absence of a platelet defect in *RhoF^−/−^* mice is not due to increased expression of other small GTPases. Irrespective of this, the presence of other Rho GTPases in platelets, Cdc42 in particular provides the possibility that the role(s) of Rif may be functionally redundant with other small G-proteins. It is known that Rif and Cdc42 share downstream effector proteins, including mDia1 and mDia2 and as such the absence of Rif does not preclude activation of these effectors by Cdc42.

### *RhoF* ^−/−^ Platelets Adhere and form Filopodia in Whole Blood under High Shear

**Figure 7:**
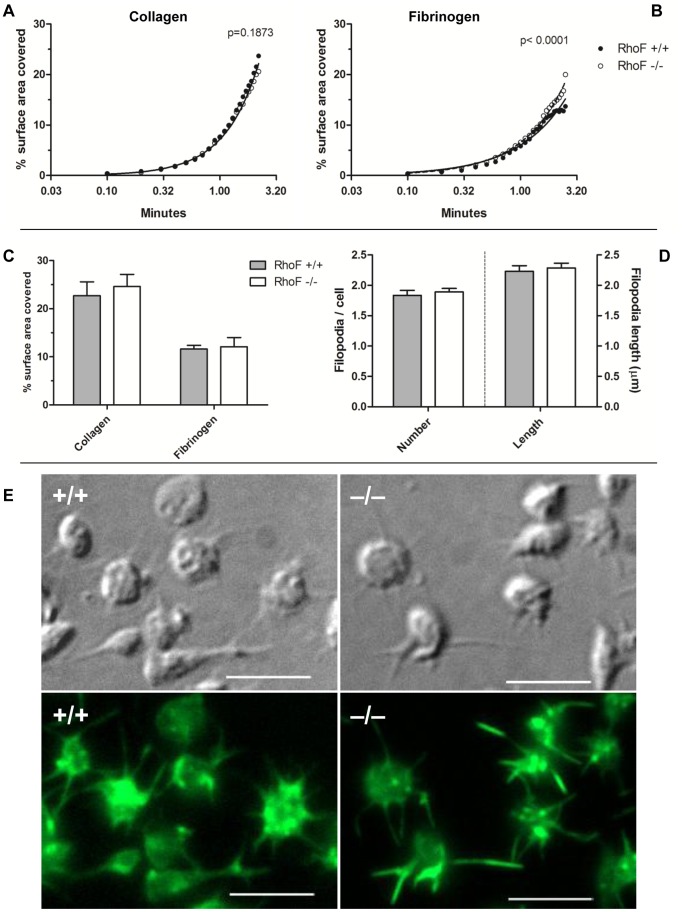
Analyses of in vitro flow assay video frames at 6 second intervals. Data points represent mean values of at least 8 mice per group. Analysis of adherence of platelets to immobilized collagen (**A**) indicates that accumulation of *RhoF^−/−^* platelets on collagen follows a similar time course to wild-type controls, suggesting that Rif is not required for initial adhesion events to collagen. (**B**) Video analyses of *in vitro* flow over fibrinogen identified a difference in the pattern of *RhoF^−/−^* platelet accumulation compared with wild type controls. (**C**) After a 3-minute wash at 1000 s^−1^ with buffer to remove erythrocytes and non-stably adhered platelets, 30 random images were taken and the surface area covered by platelets analysed. *RhoF^−/−^* mice form stable thrombi on both collagen and fibrinogen surfaces to a comparable degree to wild-type controls. Data are presented as mean ± SEM of at least 8 mice per group. (**D**) *RhoF^−/−^* platelets form actin-containing filopodia on fibrinogen surfaces under arterial shear conditions comparably with wild-type controls. Following in vitro flow of whole blood over fibrinogen coated surfaces, the number of filopodia per platelet and the length of filopodia were analyzed on 100 cells. Values are expressed as mean filopodia per cell and mean filopodia length ± SEM of 3 mice per group. (**E**) Post-washing, adherent cells were fixed with 500 µl 4% PFA flowed over surfaces at 1000 s^−1^, coverslips removed from flow chambers, permeabilized, stained with FITC-phalloidin and imaged by fluorescence and DIC microscopy. Identical parts of the images in both DIC and fluorescence channels are presented such that cell surface structures and the actin cytoskeleton within them can be directly compared. Scale bars represent 5 µm.

Although platelets lacking Rif were able to form filopodia and spread under static conditions, these assays less accurately represent the physiologic situation, where platelets interact with extracellular matrix proteins under shear in the presence of erythrocytes, leukocytes and plasma proteins. The ability of *RhoF^−/−^* platelets to adhere to fibrillar collagen and fibrinogen under flow was therefore tested in whole blood using a parallel plate flow chamber assay. Under non-coagulating conditions, *RhoF^−/−^* platelets adhered to collagen coated surfaces at an equivalent rate to wild-type controls (Fig. 7A). On fibrinogen coated surfaces *RhoF^−/−^* platelets adhered at a significantly different rate to wild-type platelets (p<0.0001) by extra sum-of-squares F-test (Fig. 7B). The physiological significance of this difference is unclear. The observed difference may imply that Rif plays a negative regulatory role in the interactions between platelets and fibrinogen. The results of other assays of inside-out and outside-in signalling through the fibrinogen receptor in *RhoF^−/−^* platelets do not support such a conclusion however. Although there was a statistically significant difference in the rate of *RhoF^−/−^* platelet adherence to fibrinogen compared with wild-types, after washing the surface area covered with stably adherent thrombi was equivalent in Rif deficient samples and wild-type controls on both collagen and fibrinogen coated surfaces (Fig. 7C). Correlative DIC and FITC-phalloidin stained microscopy images of fixed platelets post-flow indicate that *RhoF^−/−^* platelets are able to form actin-containing filopodia on fibrinogen surfaces during adherence at arterial shear, and that platelets lacking Rif form filopodia of comparable lengths in similar numbers to wild-type controls (Fig. 7D,E).

### Conclusions

Evaluations of multiple aspects of platelet function including assessments of inside-out and outside-in signaling through integrin α_IIb_β_3_ and secretion from alpha and dense granules have not identified an essential role for Rif in mouse platelets. In addition our data suggest Rif is not required for the actin rearrangements in megakaryocytes necessary for thrombopoiesis. Most importantly, static adhesion and flow chamber assays suggest that Rif is not essential for platelet filopodia formation or for manipulating the actin cytoskeleton during other platelet shape change events. As such, the question of which Rho GTPase(s) regulate platelet filopodia formation remains open. It should be noted however that lack of an identified role for Rif in mouse platelets does not preclude a role for this protein in human platelets.

There are two possible explanations for the lack of an observed phenotype in the *RhoF^−/−^* mice. The first is that Rif genuinely has no role in platelet function. If that is the case however, its continued expression in platelets would not appear to be cost-effective in evolutionary terms. The second possibility and the one we consider probable, is that Rif plays redundant roles in platelets for which other Rho GTPases can substitute when Rif is absent. Our *RhoF^−/−^* mouse model only enables us to identify non-redundant roles for this protein in platelets, such that we cannot differentiate a situation where Rif plays no role at all from the scenario where it only has overlapping functions with other Rho GTPases. Phylogenetic analysis of Rho GTPases suggests that the functions of Rif are most likely to overlap with RhoD [24]. Analysis of platelet mRNA sequences [11] suggests that RhoD is not expressed however and our own attempts to identify RhoD at the protein level by immunoblotting support this (data not shown). It is likely that Cdc42 is at least partly responsible for filopodia formation in platelets. The full extent of the role of Cdc42 in platelets is unclear due to discrepancies in the results of two publications employing distinct gene deletion strategies [7], [25]. As mentioned previously, both Rif and Cdc42 are known to activate effector proteins such as formins [26] to enable cells to produce structures through multiple mechanisms and to fine-tune the final phenotype to best suit the environmental requirements [27]. Interestingly, a recent study of platelets in the *Dia1^−/−^* mouse [28] found no defects in clot retraction or in fibrinogen binding, P-selectin surface expression, or cell spreading in response to either CRP or thrombin stimulation. As such, data from the *Dia1^−/−^* mouse is consistent with our analyses of *RhoF^−/−^* platelets, which suggests pathways other than Rif-mDia1 regulate filopodia formation in platelets. Alternative mechanisms which remain to be investigated include known interactors of Rho GTPases such as the multidomain membrane deforming protein IRSp53 [29].

In order to test whether Cdc42 is compensating for Rif in the *RhoF^−/−^* mice, strategies to inhibit or prevent the function of Cdc42 could be employed. Pharmacologic inhibition of Cdc42 with compounds such as secramine A [30] has previously been used to investigate the role of Cdc42 in platelets [31]. Although this could be undertaken in the *RhoF^−/−^* mouse, the selectivity of secramine A has been questioned [7], which would significantly impact on its utility for this purpose. The preferable alternative would be genetic manipulation to produce mouse platelets devoid of both Rif and Cdc42. This is no small undertaking however since *Cdc42^−/−^* platelets can only be produced through conditional targeting because constitutive *Cdc42* null mice are not viable. Although challenging to undertake, such follow-up work may be necessary to identify the critical regulators of filopodia formation by platelets and thereby clarify the mechanisms by which platelets generate structures vital for their hemostatic function.

## Supporting Information

Figure S1 Aliquots of washed platelets were stimulated with various concentrations of thrombin for 10 minutes **(A)** or with 1 U/ml thrombin for various time points **(B)**. Platelets were then fixed, permeabilized and stained with FITC-phalloidin prior to analysis by flow cytometry to provide an estimate of cellular F-actin content. Data are expressed as percentages of basal median fluorescent intensity and are presented as mean ± SEM for at least 5 mice per group. P values represent the significance of the comparison between the best-fit curves for the two datasets by extra sum-of-squares F-test.
(TIF)

Video S1 Real-time visualisation of wild-type platelets adhering to and spreading on a collagen related peptide coated surface, demonstrating filopodia and lamellipodia formation. Live cell differential interference contrast imaging was performed using multichamber plastic slides. Platelets were added to wells containing Tyrode’s-HEPES buffer at 37°C to a final concentration of 3×10^7^ cells/ml. Images were collected at 1 frame/s for 20 minutes. These images were then compiled into video files using ImageJ.
(AVI)

Video S2 Real-time visualisation of *RhoF^−/−^* platelets adhering to and spreading on a collagen related peptide coated surface, demonstrating filopodia and lamellipodia formation. Live cell differential interference contrast imaging was performed using multichamber plastic slides. Platelets were added to wells containing Tyrode’s-HEPES buffer at 37°C to a final concentration of 3×10^7^ cells/ml. Images were collected at 1 frame/s for 20 minutes. These images were then compiled into video files using ImageJ.
(AVI)

Video S3 Real-time visualisation of wild-type platelets adhering to and spreading on a fibrinogen coated surface, demonstrating filopodia and lamellipodia formation. Live cell differential interference contrast imaging was performed using multichamber plastic slides. Platelets were added to wells containing Tyrode’s-HEPES buffer at 37°C to a final concentration of 3×10^7^ cells/ml. Images were collected at 1 frame/s for 20 minutes. These images were then compiled into video files using ImageJ.
(AVI)

Video S4 Real-time visualisation of *RhoF^−/−^* platelets adhering to and spreading on a fibrinogen coated surface, demonstrating filopodia and lamellipodia formation. Live cell differential interference contrast imaging was performed using multichamber plastic slides. Platelets were added to wells containing Tyrode’s-HEPES buffer at 37°C to a final concentration of 3×10^7^ cells/ml. Images were collected at 1 frame/s for 20 minutes. These images were then compiled into video files using ImageJ.
(AVI)

Video S5 Real-time visualisation of wild-type platelets adhering to and spreading on a von Willebrand factor coated surface in the presence of botrocetin, demonstrating filopodia and lamellipodia formation. Live cell differential interference contrast imaging was performed using multichamber plastic slides. Platelets were added to wells containing Tyrode’s-HEPES buffer at 37°C to a final concentration of 3×10^7^ cells/ml. Images were collected at 1 frame/s for 20 minutes. These images were then compiled into video files using ImageJ.
(AVI)

Video S6 Real-time visualisation of *RhoF^−/−^* platelets adhering to and spreading on a von Willebrand factor coated surface in the presence of botrocetin, demonstrating filopodia and lamellipodia formation. Live cell differential interference contrast imaging was performed using multichamber plastic slides. Platelets were added to wells containing Tyrode’s-HEPES buffer at 37°C to a final concentration of 3×10^7^ cells/ml. Images were collected at 1 frame/s for 20 minutes. These images were then compiled into video files using ImageJ.
(AVI)
