# Supplementary figures and images for: The Small GTPase Rif Is Dispensable for Platelet Filopodia Generation in Mice
Source: PLoS One. 2013 Jan 24;8(1):e54663. doi: 10.1371/journal.pone.0054663 (PMC3554654; doi:10.1371/journal.pone.0054663)

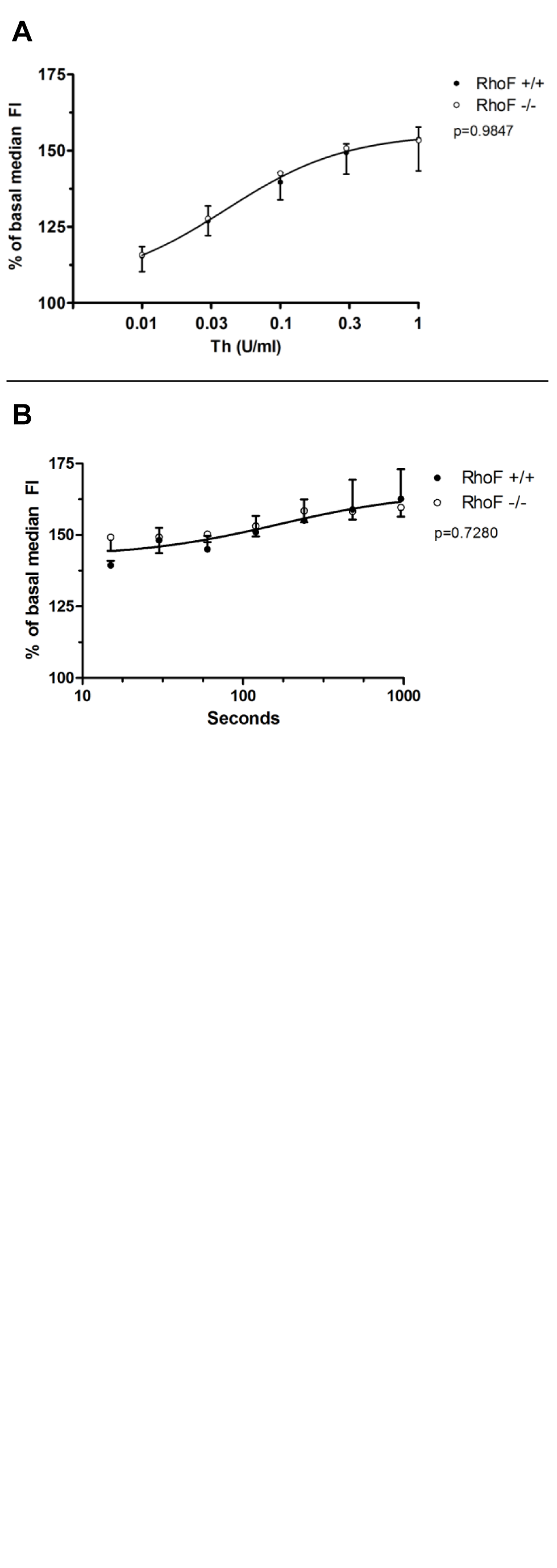

Supplement: Figure S1 — Aliquots of washed platelets were stimulated with various concentrations of thrombin for 10 minutes (A) or with 1 U/ml thrombin for various time points (B). Platelets were then fixed, permeabilized and stained with FITC-phalloidin prior to analysis by flow cytometry to provide an estimate of cellular F-actin content. Data are expressed as percentages of basal median fluorescent intensity and are presented as mean ± SEM for at least 5 mice per group. P values represent the significance of the comparison between the best-fit curves for the two datasets by extra sum-of-squares F-test. (TIF) [file pone.0054663.s001.tif]
